# Protein Phosphatase (*PP2C9*) Induces Protein Expression Differentially to Mediate Nitrogen Utilization Efficiency in Rice under Nitrogen-Deficient Condition

**DOI:** 10.3390/ijms19092827

**Published:** 2018-09-19

**Authors:** Muhammad Waqas, Shizhong Feng, Hira Amjad, Puleng Letuma, Wenshan Zhan, Zhong Li, Changxun Fang, Yasir Arafat, Muhammad Umar Khan, Muhammad Tayyab, Wenxiong Lin

**Affiliations:** 1 Key Laboratory for Genetics, Breeding and Multiple Utilization of Crops, Ministry of Education/College of Crop Sciences, Fujian Agriculture and Forestry University, Fuzhou 350002, China; lizhong021@126.com (Z.L.); fcx007@fafu.edu.cn (C.F.); tyb.pk@hotmail.com (M.T.); 2 Fujian Provincial Key Laboratory of Agroecological Processing and Safety Monitoring, College of Life Sciences, Fujian Agriculture and Forestry University, Fuzhou 350002, China; heraamahmood@yahoo.com (H.A.); pulengletuma@yahoo.com (P.L.); arafat_pep@yahoo.com (Y.A.); umar.khan018@yahoo.com (M.U.K.); 3 Key Laboratory of Crop Ecology and Molecular Physiology (Fujian Agriculture and Forestry University), Fujian Province University, Fuzhou 350002, China; M18120821563@163.com (S.F.); 1170525027@fafu.edu.cn (W.Z.)

**Keywords:** N utilization efficiency, proteomics, 2D, protein phosphatase, rice isogenic line, SnRK1, 14-3-3

## Abstract

Nitrogen (N) is an essential element usually limiting in plant growth and a basic factor for increasing the input cost in agriculture. To ensure the food security and environmental sustainability it is urgently required to manage the N fertilizer. The identification or development of genotypes with high nitrogen utilization efficiency (NUE) which can grow efficiently and sustain yield in low N conditions is a possible solution. In this study, two isogenic rice genotypes i.e., wild-type rice kitaake and its transgenic line *PP2C9TL* overexpressed protein phosphatase gene (*PP2C9*) were used for comparative proteomics analysis at control and low level of N to identify specific proteins and encoding genes related to high NUE. 2D gel electrophoresis was used to perform the differential proteome analysis. In the leaf proteome, 30 protein spots were differentially expressed between the two isogenic lines under low N level which were involved in the process of energy, photosynthesis, N metabolism, signaling, and defense mechanisms. In addition, we have found that protein phosphatase enhances nitrate reductase activation by downregulation of SnRK1 and 14-3-3 proteins. Furthermore, we showed that *PP2C9TL* exhibits higher NUE than *WT* due to higher activity of nitrate reductase. This study provides new insights on the rice proteome which would be useful in the development of new strategies to increase NUE in cereal crops.

## 1. Introduction

The present capacity to provide food for the increasing global population is due to the green revolution, which is based on the adoption of semidwarf cereals with high yield. However, to achieve an increase in crop production depends on the application of nitrogen (N) fertilizers [1,2,3]. Application of N fertilizer has become a key factor to improve the crop productivity. Unfortunately, the extensive use of N fertilizers is causing harm to the soil as well as water bodies. Nitrogen leached from agricultural lands, in the form of nitrate causing eutrophication in rivers, lakes, and oceans, is decreasing aquatic diversity and damaging drinking water [4]. Therefore, it is urgently required to limit or reduce the application of fertilizers without affecting crop production [5,6]. Nitrogen (N) is an essential plant macronutrient as it is the fundamental element of plant components such as nucleic acids, amino acids, proteins, enzymes, chlorophyll, and various hormones. Availability of N plays a vital role in plant growth, senescence, flowering time, photosynthesis, and translocation of photosynthates [7]. Worldwide consumption of N-based fertilizers is approximately 119.40 million tons with an annual growth of 1.4% [8]. Asia uses 62.1% of the total nitrogenous fertilizers and China alone shares 18% of the Asian N consumption [8]. However, it is alarming that major cereal crops like wheat, rice, and maize only utilize 30–40% of the applied N. The remaining unutilized 60–70% of applied N causes severe health and environmental risks [9]. 

Improving the N use efficiency (NUE) in crops would be helpful to increase crop production without any penalty to the environment. It is estimated that 1.1 billion USD can be saved by increasing 1% NUE and can also help reduce environmental pollution [10]. Several approaches from agronomic methods to transgenic efforts have been tried to solve this issue such as the split application of N, use of nitrification inhibitors, and the slow release of fertilizers [11,12]. Conventional procedures such as selective breeding improve grain yield heritability [13]. However, it cannot explain the genetic basis for the improvements of complex quantitative traits like NUE [14]. Scientists have developed gene-overexpressed mutants to increase the biomass and plant N contents attempting to enhance NUE in crop production [15,16,17]. The overexpression of high-affinity ammonium transport system (HATS) like NRT2.1 increases the nitrate influx, but there was no improvement in the phenotypic NUE [18,19]. However, the overexpression of nitrite reductase (NiR) and nitrate reductase (NR) in tobacco and Arabidopsis decreases nitrate levels in plant tissues but fail to improve the biomass or grain yield [12,20]. The ectopic expression of cytoplasmic glutamine synthetase1 (GS1) [21] and glutamine synthetase 2 (GS2) [22,23] have some positive effects on plant biomass and grain yield. Studies suggest that targeted manipulation of one gene or just one component of the N signaling pathway may not be enough to significantly improve overall NUE because it is a quantitative trait controlled by many genes and interacting regulatory pathways that are linked with the actual NUE of a crop plant [24]. 

Many scientists have tried to exploit the regulatory pathways involved in the transport of nitrate in plant organs. The reversible proteins phosphorylation by protein phosphatases is an essential mechanism to regulate the different biological processes. In plants, PP2Cs is a major phosphatase-encoding gene family that has emerged as a key regulator of stress signaling [25,26]. Protein phosphatases (PP2Cs) negatively regulate abscisic acid (ABA) signaling. In Arabidopsis, PP2C proteins such as ABA-insensitive 1 (ABI1), ABI2, and Hypersensitive to ABA 1 (HAB1) have been identified to regulate ABA-induced signaling under biotic and abiotic stresses by interacting with SnRK2s and PYR/PYL/RCARs [27,28,29,30,31]. The activity of nitrate transporters (NPF6.3) is regulated by CBL9 (Calcineurin B like protein 9) and CIPK23 complex (CBL interacting protein kinase 23), CBL9 phosphorylates the CIPK23 activating the protein complex. The activated CIPK23 inhibits the activity of NPF6.3 [32]. However, protein phosphatase (PP2C9s) enhances the NPF6.3-dependent nitrate sensing by dephosphorylating the CIPK23 and CBL9 complex, nitrate signaling, and nitrate transport [33]. Recently, scientists have started to focus on the critical role of protein phosphates in NUE. In our preliminary study, we have found that the protein phosphatase (PP2C9) was closely related with the improvement of the NUE in rice by enhancing N uptake and assimilation, however, the appropriate information still lacks, especially regarding the PP2C9 regulatory mechanism for high NUE in rice plants. 

In order to exploit the regulatory role of protein phosphatase (PP2C9) regarding NUE, we used the transgenic japonica rice line overexpressing the protein phosphatase (PP2C9) for differential proteomics analysis to identify the genes and signaling pathways mediated by PP2C9 under nitrogen limiting conditions. Although the plant organs contain the same complement of the genome, the expression of genes and proteins accumulation varies widely. Proteomic studies can overcome the limitations of post-translational modifications that occur during DNA/RNA transcription and expression processes, and provide proper information about plant biological functions at a particular time course. Rice (*Oryza sativa* L.) a staple food for half of the population worldwide and of immense agricultural importance, has been a popular research subject for agriculture scientists [34,35]. In China, by 2030, rice demand will have increased up to 14%, to fulfill the increasing demand farmers are extensively using N fertilizers [36,37]. The amount of N fertilizer used (209 kg ha^−1^) for rice production in China is 90% more than the global use [38], and this makes China the world leading N fertilizer consumer with low N utilization efficiency [39,40] and high environmental risk. Considering all these issues, we have developed isogenic lines including the transgenic rice line overexpressing protein phosphatase (*PP2C9TL*) and its wild-type (kitaake); we used these materials to investigate the underlying mechanism associated with NUE in rice exposed to a limited nitrogen supply condition through differential proteomics. We have identified that the protein phosphatase (PP2C9) gene, which functions to significantly improve the NUE by enhancing N uptake and assimilation by regulating nitrate reductase activation via dephosphorylation of SnRK1 and 14-3-3 proteins.

## 2. Results

### 2.1. Physiological Performance of PP2C9TL and WT

**Figure 1:**
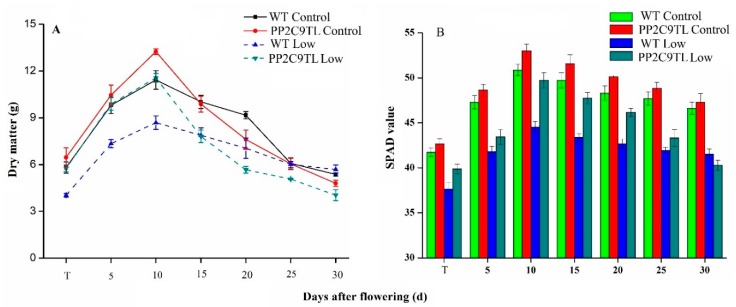
(**A**) The leaf dry matter (g) of *PP2C9TL* and *WT* at tillering (T), 5, 10, 15, 20, 25, and 30 DAF. (**B**) The chlorophyll contents of *PP2C9TL* and *WT* at tillering (T), 5, 10, 15, 20, 25, and 30 DAF.

The present study shows that the overexpression of protein phosphatase (*PP2C9*) significantly improves rice plant performance under N deficient conditions. We measured the dry matter of leaf from the tillering (T) to maturity stage of rice plants which were sampled in the time courses at 5 days after flowering (DAF), 10 DAF,15 DAF, 20 DAF, 25 DAF, and 30 DAF, based on our initial findings [41]. As compared to *WT*, in *PP2C9TL* the dry matter of leaf significantly increased up to 10 DAF, after that it slowly started to decrease from 15 DAF to 20 DAF and finally, a significant decline was observed from 20 DAF up to 30 DAF under control and N deficient conditions, as shown in (Figure 1A, Figures S1 and S2). In *WT*, the dry leaf matter increased up to 10 DAF and then insignificantly decreased from 15 DAF to 30 DAF. We found that *PP2C9TL* efficiently tolerated N stress and produced the almost same amount of dry matter at low N as the *WT* produced at the control level of N, whereas, low N stress affected the *WT* plants’ growth and decreased the leaf biomass as shown in Figure 1A. Similarly, higher chlorophyll content was observed in *PP2C9TL* than *WT.* We used the SPAD meter to measure the chlorophyll contents. The chlorophyll content increased from T to 10 DAF and decreased at 30 DAF in both *WT* and *PP2C9TL*, as shown in (Figure 1B, Figures S1 and S2). The physiological indices showed that *PP2C9TL* could effectively increase dry leaf matter and photoassimilates, which could then be transported to grain to increase the yield.

### 2.2. NUE and Yield Performance of PP2C9TL and WT 

**Figure 2:**
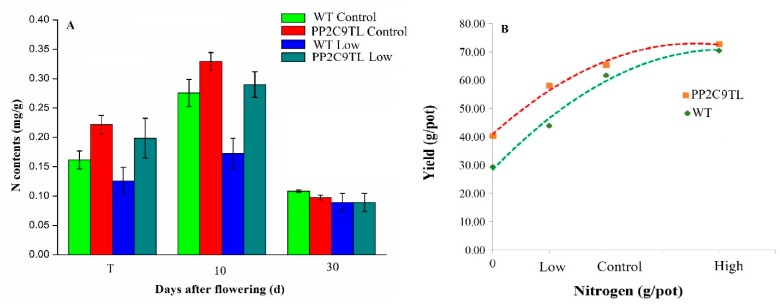
(**A**) The leaf N contents of *PP2C9TL* and *WT* at tillering T, 10 DAF, and 30 DAF; (**B**) the NUE of *WT* and *PP2C9TL* at low, control, and high levels of N.

In order to estimate the nitrogen uptake and NUE, we have calculated the leaf N content in *PP2C9TL* and *WT* in control and low N conditions. The significant differences in N content of the leaf were found at 10 DAF between the two genotypes under low N conditions. The *PP2C9TL* showed efficient N uptake compared to *WT* (Figure 2A). The highest difference in leaf N contents between the two genotypes was found at the heading stage (10 DAF). The grain yields across different levels of N are shown in Figure 2B. The difference between yields of *PP2C9TL* and *WT* is higher without N and with a low level of N, whereas, the yield differences decreased with the increase in N level. *PP2C9TL* produced a higher yield (58 g/pot) than *WT* (43 g/pot) at low N condition. The yield of *PP2C9TL* in low N level is almost equal to *WT* yield at the control level of N, these results indicate that *PP2C9TL* produced a high yield by consuming less N. The differences in yield at given levels of N confirmed the genetic variation for NUE between the two genotypes. Physiological attributes showed significant differences at 10 DAF between the two genotypes, clearly the *PP2C9TL* performed better than the *WT* so, to investigate the molecular pathway involved for higher NUE in *PP2C9TL* at low N, we designed differential proteomic experiments at 10 DAF.

### 2.3. Leaf Proteome Analysis of the Two Isogenic Lines under N Deficient Conditions

**Figure 3:**
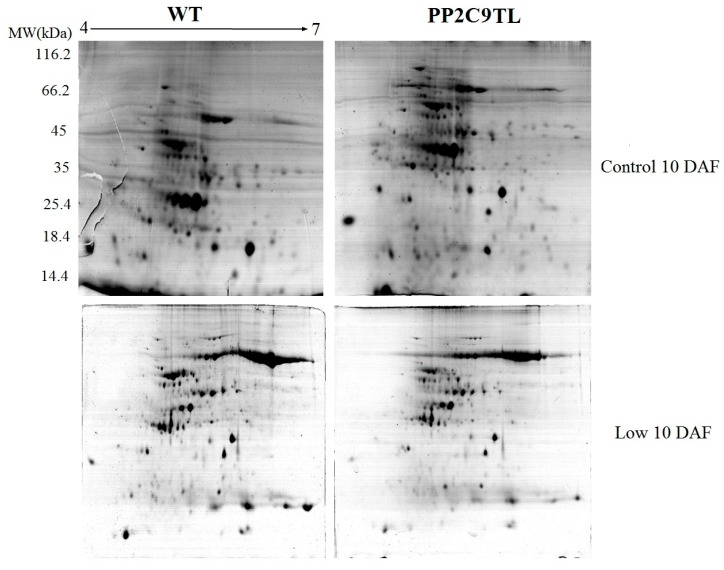
Representative 2-DE gel electrophoresis images of leaf proteins from the *WT* and *PP2C9TL* at 10 DAF under control and low N conditions. The MW (kilodaltons) and pI of the proteins are shown on the left and at the top, respectively.

**Figure 4:**
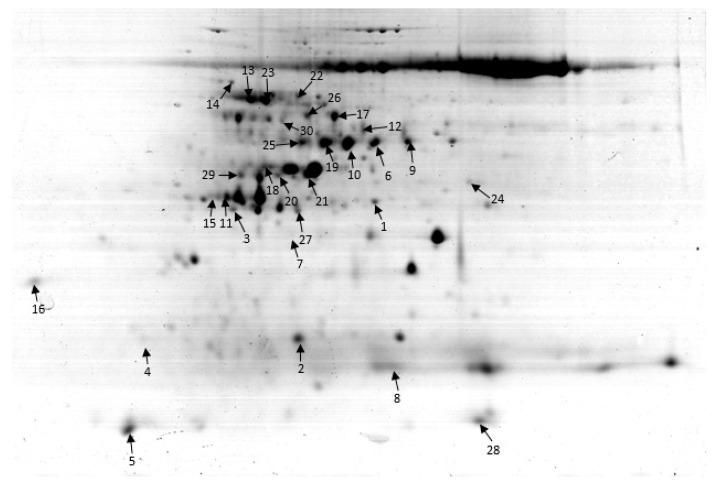
A representative 2-D gel electrophoresis image showing total differentially expressed protein spots in the leaf under low N condition.

Differential proteomics of leaves from both *PP2C9TL* and its wild-type, *Kitaake japonica* rice, in low nitrogen conditions, lead to the identification of proteins involved in regulation of N uptake. Representative gels were shown in Figure 3, Figures S3 and S4. A total of 30 protein spots were found to be differentially expressed between the pH ranges of 4 to 7 (Figure 4, Figures S5 and S6). Imagemaster 5.0 was used for expression abundance of protein spots based on their relative volume. However, both genotypes showed differential protein expression under the N deficient condition. 

### 2.4. Functional Characterization of the Identified Proteins

**Figure 5:**
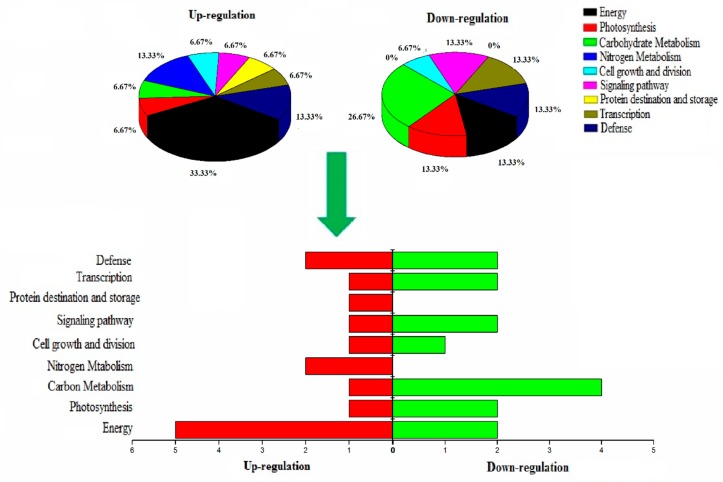
Pie graph percentage distribution display of the identified proteins described in (Table 1) based on biological function along with sidewise bar graph depicting up-and down-regulating proteins.

The 30 proteins showed differential expression in the leaf (Table 1). Among these identified proteins, 14 (47%) were upregulated and 16 (53%) were downregulated. Web gene ontology annotation plot (WEGO) analysis was carried out to identify the biological function and cellular location of these proteins. These identified proteins are divided into nine groups according to their molecular and biological function: energy (23.33%), carbohydrate metabolism (16.67%), defense (13%), signaling (10%), transcription (10%), nitrogen metabolism (6.67%), cell growth and division (6.67%), and protein folding and storage (3.33%), as shown in Figure 5.

### 2.5. Subcellular Characterization of the Identified Proteins 

**Figure 6:**
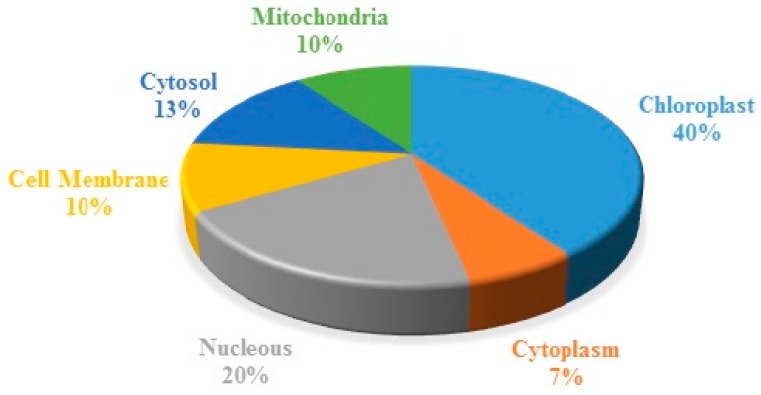
Subcellular indexing of the identified proteins.

Almost all the organelle functions were affected by low N as the identified proteins belong to different organelles. The expressed proteins were mostly associated to chloroplast (40%) followed by nucleus (20%), cytosol (10%), mitochondria (10%), cell membrane (10%), and cytoplasm (7%) (Figure 6). 

### 2.6. Potential Molecular Pathway Based on Differentially Expressed Proteins

**Figure 7:**
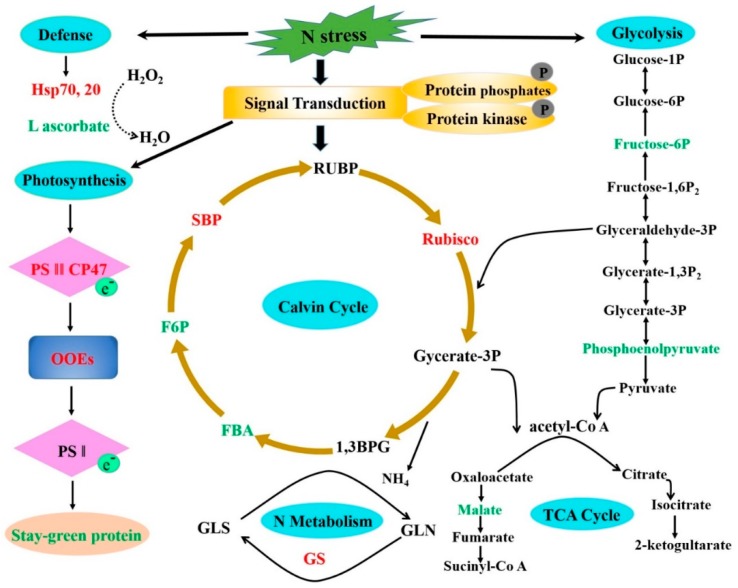
Potential molecular pathway based on differentially expressed proteins in *PP2C9TL* under N deficient conditions. The red color text shows the upregulation and green color text shows downregulation of proteins. Note RUBP (Ribulose 1, 5-bisphosphate), RuBisCO (Ribulose-1,5-bisphosphate carboxylase/oxygenase), 1,3 BPG (1,3-Bisphosphoglycerate), FBA (Fructose-bisphosphate aldolase), F6P (Fructose-bisphosphate aldolase), SBP (sedoheptulose-1,7-bisphosphatase), PSII (Photosystem II), OOEs (oxygen evolving enhancer proteins), PSΙ (Photosystem Ι), GLS (Glutaminase), and GS (Glutamate synthase).

**Figure 8:**
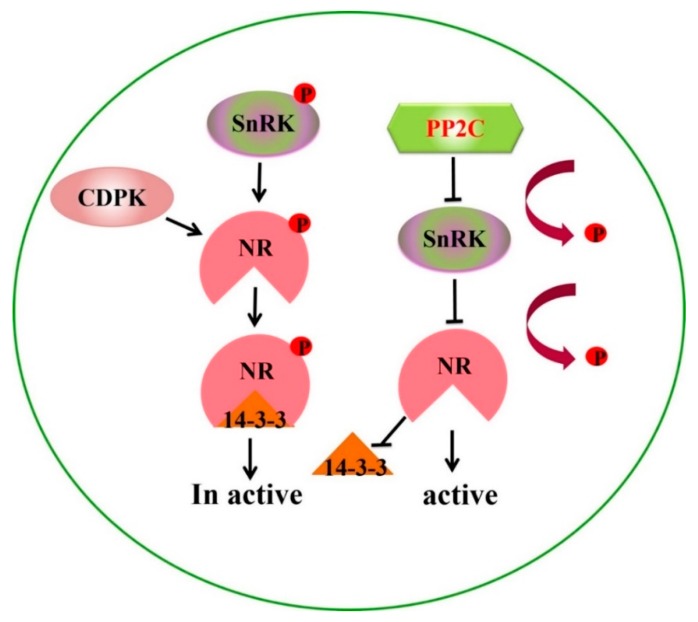
*PP2C9* role for nitrate reductase activation by dephosphorylation of SnRK. 14-3-3 proteins SnRK (SNF1-related kinase) and CDPK (calcium-dependent protein kinases) can phosphorylate NR in crop plants. After phosphorylation of NR, it binds with the 14-3-3 protein to form a complex which is inactive or has low activity. *PP2C9* interacts with 14-3-3 and SnRK and dephosphorylates them to activate NR.

The differential expression of proteins showed that low N influenced different molecular pathways involved in photosynthesis, Calvin cycle, glycolysis, N metabolism, and defense mechanism. The *PP2C9TL* efficiently tolerate the N stress than *WT.* In *PP2C9TL*, the RuBisCO (Ribulose-1, 5-bisphosphate carboxylase/oxygenase), Photosystem II, and oxygen evolving enhancer proteins (OOEs) were upregulated compared to *WT*, and the stay green protein was downregulated. The photosystem II and RuBisCO helps *PP2C9TL* to maintain photosynthesis during N deficiency better than the *WT.* RuBisCO plays an important role in the Calvin cycle to generate energy in *PP2C9TL* and is interlinked with the glycolysis and TCA cycle, as shown in Figure 7. However, in *PP2C9TL* the overexpressed PP2C9 gene takes part in the activation of nitrate reductase (NR) via dephosphorylation of 14-3-3 and SnRK, as shown in Figure 8. The differential expression showed that 14-3-3 and SnRK are downregulated in *PP2C9TL*. The higher activity of NR in *PP2C9TL* generates more nitric oxide which increases N uptake by enhancing the development of lateral roots. 

### 2.7. Western Blotting of the Important Differentially Expressed Protein

**Figure 9:**
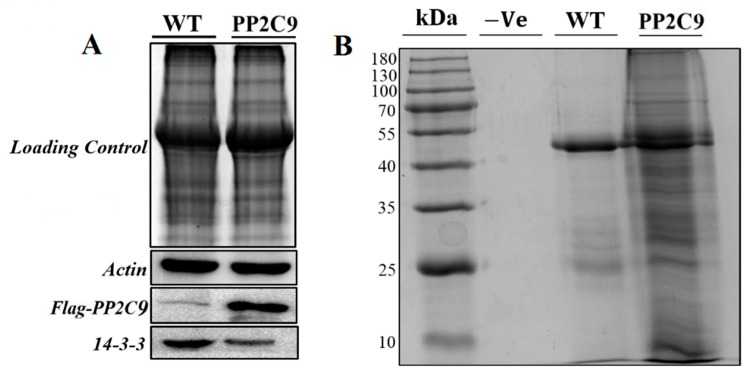
(**A**)Western blotting of differentially expressed proteins in *PP2C9TL* and *WT* at 10 DAF stage. The top photo shows the SDS-PAGE separation of two protein samples used as loading control. The bottom photo shows gel transferred onto nitrocellulose membrane for western blotting to detect actin, anti-Flag-*PP2C9*, and 14-3-3 protein for *WT* and *PP2C9TL* at the 10 DAF stage. (**B**) Co-immunoprecipitation (Co-IP) assays of *PP2C9* associated protein analyzed by SDS-PAGE. Protein molecular weight marker, negative control without antibodies, *WT* protein, and the third is the *PP2C9TL* protein. 2.8. Identification of PP2C9 Associated Protein by Co-Immunoprecipitation (CO-IP) and Mass Spectrometry.

The 14-3-3 protein is a highly conserved protein in crop plants and is directly involved in the NR inactivation mechanism. So Western blotting was carried out to verify the *PP2C9TL* and the differential expression of 14-3-3 protein detected by 2DE gels using primary mouse antisera and the HRP-anti-mouse IgG as the secondary antibodies against Flag-tag PP2C9 and 14-3-3. The Western blot results confirmed the downregulation of 14-3-3 in *PP2C9TL*, whereas, the presence of Flag-tag verified the enhanced expression of *PP2C9* in *PP2C9TL*, as shown in Figure 9A.

In order to find the PP2C9-associated protein interactions and to pull out all protein complexes among *PP2C9TL* and *WT*, we carried out the immunoprecipitation assay by using the (rabbit antisera Flag-tag antibodies). The immunoprecipitation assay results showed that *PP2C9TL* had obvious differences in bands compared with *WT.* These differential protein bands were located at 20 to 80 kDa as shown in Figure 9B. Protein bands were excised and exposed to digestion and analysis followed by tandem mass spectrometry (MS/MS). The identified proteins by (MS/MS) analysis were shown in Table 2. The Co-IP validates the results obtained by comparative proteomics as most of the identified proteins were similar with 2DE proteins and involved in photosynthesis, energy, N metabolism, signaling cascades, and defense mechanisms. So these identified differential proteins from Co-IP and 2DE were responsible for higher NUE of *PP2C9TL* than *WT*.

## 3. Discussion

### 3.1. Regulatory Role of PP2C9 for Higher NUE in PP2C9TL under N Deficient Conditions

The reversible proteins phosphorylation by protein phosphatases is an essential mechanism to regulate the different biological processes. In plants, PP2C9s are a major phosphatase-encoding gene family and have emerged as key regulators of stress signaling. To gain a more comprehensive understanding of the function of PP2C9 in response to N stress, we devised a comparative proteomics strategy, with this approach, we identified three regulatory proteins the PP2C9, 14-3-3 family, and SnRK. PP2C9 deals with phosphorylation/dephosphorylation of several proteins, so it is quite possible to alter the signaling pathways. The proteins involved in signaling (14-3-3 Spot 15 and Beta subunit 2 of SnRK1 Spot 27) were downregulated in *PP2C9TL* compared with that in *WT*, whereas the protein phosphatase 2C 16 (Spot 25) was upregulated in *PP2C9TL*. The upregulation of protein phosphatase 2C 16 is not surprising as it is overexpressed in *PP2C9TL*. The downregulation of SnRK1 was also observed in Arabidopsis during N and K stress [42]. 14-3-3 was reported to be a key regulator of nitrogen and carbon metabolism through interaction with multiple signal transduction pathways [43]. In Arabidopsis, the overexpression of 14-3-3 decreased the sugar- and N-based compounds and also reduced the levels of malate and citrate, which are the intermediate compounds of the TCA cycle [44]. In *PP2C9TL*, the 14-3-3 protein was downregulated, which might be due to the overexpressed *PP2C9* gene which dephosphorylates the 14-3-3, the downregulation of 14-3-3 was confirmed by the Western blot results. *PP2C9* interacts with 14-3-3 and SnRK1 and dephosphorylates them to activate NR. SnRK1 and CDPK (calcium-dependent protein kinases) can phosphorylate NR in crop plants [45,46,47]. Phosphorylation itself does not alter NR activity. However, 14-3-3 proteins and cations, such as Mg^2+^, are present, and this complex of phosphorylated NR shows low activity, whereas *PP2C9* reactivates NR by dephosphorylation [48,49]. Our results suggest that the 14-3-3s regulate the C and N metabolism through interaction with SnRK1 and PP2C9. NR activity is most important for nitrogen uptake and assimilation is the most probable reason for higher NUE of *PP2C9TL* in low nitrogen.

### 3.2. The Physiological Basis for NUE and Grain Yield

NUE of crop plants roughly depends upon two factors. First, how efficiently a plant absorbs N from the soil (N uptake efficiency) and second, how efficiency can the plant utilize this N to produce a high grain yield [50]. Whatever the N level, the high vegetative growth at flowering stage was favorable for higher N uptake efficiency. The N uptake at flowering was significantly correlated with grain yield at both a low and high level of N, especially at low N where it determines seed number [20,51]. Grain yield depends upon complex biochemical processes, especially C and N interactions, which lead to the production of dry matter [52]. In this study, *PP2C9TL* have higher vegetative growth at the flowering stage. *PP2C9TL* increased leaf dry matter and N contents at 10 DAF and thereafter, consequently decreased until 30 DAF. This might occur because the proteins, fats, carbohydrates, minerals, and vitamins are transported and accumulated into the grain from vegetative parts that ultimately reduce the dry weight of plants. The relationship between source–sink is the key factor for the grain yield of cereal crops, the plant leaves are the primary source of photoassimilates, whereas, the grains are the primary sink [53]. Our results are consistent with previous findings that a positive correlation was observed between the dry matter accumulations at the heading stage which is negatively correlated at the grain filling stage [54]. Arginine is a major storage form of N in plant organs and is well-known for N transport [55]. The *PP2C9TL* COIP results found arginase and aspartate aminotransferase enzymes suggesting the higher metabolism of arginine and aspartate which ensures efficient transport and storage of N in *PP2C9TL.* PP2C9 regulates N and C metabolism by activation of NR, sucrose phosphate synthase (SPS), and inactivation of PEPase [52]. Thus, it might be possible that PP2C9 together with SnRK are involved in the regulation of N and C metabolism-related enzymes through phosphorylation/dephosphorylation. *PP2C9TL* has a higher photosynthetic rate because photosynthesis is directly related with leaf N and chlorophyll contents which were higher in the *PP2C9TL* compared to *WT*. We further demonstrated that in *PP2C9TL* a high NUE and N uptake efficiency were observed at low level of N which decreases with the increase of N. The yield differences between two genotypes was decreased with the increase in N level, this might be due to surplus N availability which ultimately slow down or stop the increase in yield and reduces NUE. Extensive N application usually resulted in unlimited N absorption, lodging and yield reduction [56]. The NUE is also govern by the rice genotypes as the yield differences at same level of N depicts that two genotypes exhibit the genetic variability for NUE, this genetic variability could be helpful for crop breeders to develop the new rice genotypes with higher NUE. 

### 3.3. Proteins Expression Involved in Energy of PP2C9TL and WT Genotypes under N Deficient Conditions

As expected the proteins differential expressed under low N condition were mostly belonged to energy. RuBisCO (Ribulose-1,5-bisphosphate carboxylase/oxygenase Spot 3 and Spot 10) was downregulated under low N conditions in *WT* as compared to *PP2C9TL*. In C3 plants, about 12% of total N is consists of RuBisCO during vegetative growth so it has major significance for NUE. RuBisCO has a dual catalytic function as carboxylase for carbon dioxide assimilation and as oxygenase to trigger the photo-respiratory pathway in plants [57]. Under the low concentration of N, availability of RuBisCO was also decreased in cereals [58] and in *Arabidopsis* [59]. In *WT* RuBisCO is downregulated suggests that extensive degradation of photosynthetic apparatus [59]. However in *PP2C9TL*, this degradation is limited under low N that might be due to high uptake of N. Nitrogen influx into leaves directly determined the RuBisCO synthesis, fluctuations in RuBisCO synthesis correlate well with those for N influx throughout the leaf lifetime in rice [60]. ATP synthase and the alpha subunit of ATP synthase (Spot 20, Spot 7), mainly produced in the mitochondria or chloroplast membranes, catalyze the synthesis of ATP from ADP during energy producing biochemical cycles. In the electron transport chain of photosynthesis, the ATP synthase complex takes part in photophosphorylation of ADP to ATP providing energy to the Calvin cycle for subsequent biosynthesis [61]. In our study, under the low N condition, the concentration of this (spot 22) was higher in *PP2C9TL* than *WT*. On the other hand, the spot (7) intensity was lower in *PP2C9TL* than *WT*. The ATP synthase protein complex takes part in maintaining the function of the chloroplast during stress conditions [62]. The increased expression of ATP synthase protein in *PP2C9TL* plants provides tolerance against N deficiency; however, protein phosphatases take part in reversible phosphorylation of proteins to activate/deactivate them in different signaling pathways. In cereal crops, differential expression of ATP synthase has been observed [63]. Glycine cleavage system H protein (Spot 2), also named glycine dehydrogenase (GDC), is a multiprotein complex that is found in all living organisms. GDC is required for photorespiration in C_3_ plants, and it takes glycine, which is produced as a byproduct of the Calvin cycle, and converts it to serine through an interaction with serine hydroxyl methyltransferase (SHMT). The glycine cleavage system generates ammonia which is later assimilated by glutamine synthetase through the glutamine oxoglutarate aminotransferase cycle at the expense of one ATP and one NADPH. The intensity of this protein was higher in *PP2C9TL* than *WT*. Malate dehydrogenase (Spot 24) catalyzes the terminal step of the citric acid cycle and converts malate to oxaloacetic acid (OAA) by generating NADH. The malate dehydrogenase was downregulated in *PP2C9TL* and *WT* under low N treatment. In Arabidopsis and cereal crops, a slight upregulation of malate dehydrogenase was observed with an increase in nitrate level [63,64,65].

### 3.4. Proteins Expression Involved in Photosynthesis of PP2C9TL and WT Genotypes under N Deficient Conditions

Phosphoenolpyruvate carboxylase or PEPC primarily fixes and assimilates photosynthetic CO_2_ in C4 and crassulacean acid metabolism (CAM) plants, whereas, PEPC replenishes the TCA cycle with intermediates in the C3 plant [66,67]. PEPC was reported to be phosphorylated by the Ser/Thr kinase and PEPC kinase (PPCK), whereas, protein phosphatase 2A dephosphorylates the PEPC [68]. The PEPC (Spot 26) was found to be downregulated in the *PP2C9TL* under low N. The downregulated expression of PEPC protein in *PP2C9TL* might be due to the overexpression of *PP2C9* which ultimately deactivates the PEPC by dephosphorylation. Photosystem II CP47 reaction center protein is the constitutive transmembrane antenna proteins which interact with chlorophyll a and beta-carotene to pass the excitation energy on to the reaction center. It takes part in photosynthesis by sustaining the stability of PSII [69,70]. This protein was increased in *PP2C9TL* under low N (Spot 30). The degradation of oxygen-evolving complex (OEC) proteins was observed during low N, in response, OEC releases oxygen evolving enhancer proteins (OEEs) as a degradation product which enables the plant to adapt and survive in unfavorable conditions [71]. Oxygen-evolving enhancer proteins (OEEs) were bound to photosystem II (PSII) at the lumen side of the thylakoid membrane. The electrons generated during these reactions were transferred to photosystem I through electron transport chain which are ultimately used during NADP reduction. Electron transport from PSII to PSI, together with RuBisCO and carbon metabolism enzymes, are crucial to the photosynthetic rate [72]. In *PP2C9TL*, an increase in the OEEs helps PSII to maintain its ability to assimilate maximum photoassimilates under low N levels as reported by previous studies [73]. The stay-green protein (Spot 22) located in the thylakoid membranes triggers chlorophyll degradation during natural and dark-induced leaf senescence [74]. The stay-green protein was found to be downregulated in *PP2C9TL* relative to *WT* in low nitrogen. The downregulation of the stay-green protein shows the high tolerance ability of *PP2C9TL* in low N conditions because overexpression of stay-green protein reduced the number of lamellae in the grana thylakoids and increases chlorophyll breakdown in rice [75].

### 3.5. Proteins Expression Involved in Nitrogen Metabolism of PP2C9TL and WT Genotypes under N Deficient Conditions

In crop plants, N metabolism is most important to their nutritional availability. Two of the most important N metabolism proteins showed an upregulated expression pattern in *PP2C9TL* relative to *WT.* Nitrate reductase (NR) (Spot 23) is the most important enzyme for N metabolism and directly accounts for N assimilation rate in plants, NR is used as the main product for organ development and plant growth [76]. However, *PP2C9TL* showed an increase in expression of NR protein. We found *PP2C9TL* efficiently increased the uptake and assimilation of N under low N levels compared to the *WT.* Moreover, nitrate reductase is activated by protein phosphatase by dephosphorylation at the post-translational level. [77,78]. *PP2C9TL* overexpressed the protein phosphatase that could dephosphorylate NR to keep active on nitrate availability. Glutamine synthetase (GS) catalyzes ammonia produced by photorespiration, protein catabolism, nitrate, and ammonia metabolism and takes part in N assimilation and transportation [79,80]. Glutamine synthetase (GS) protein (Spot 19) was upregulated in *PP2C9TL* relative to *WT* during low nitrogen. This upregulated GS expression in *PP2C9TL* provides sufficient GS level to incorporate ammonia into organic compounds efficiently [81]. In the *WT* genotype, GS expression was downregulated in low nitrogen showing that N-deprived plants are undergoing more stress.

### 3.6. Proteins Expression Involved in Defense and Protein Folding of PP2C9TL and WT Genotypes under N Deficient Conditions

N stress caused upregulation of (HSP 70 Spot 17 and remorin 1 protein/Hsp20 Spot 28) in *PP2C9TL* whereas, (L-ascorbate peroxidase Spot 16 and thaumatin-like protein Spot 4) was downregulated in *PP2C9TL.* The upregulation of HSP is the most conserved adaptive strategy of plants in response to stress [82,83,84,85]. Yang et al. [86] reported an upregulation of HSP 70 in *Phaeodactylum tricornutum* under N stress. Ascorbate peroxidase (APX) exists in isoforms in different subcellular organelles such as peroxisome, chloroplasts, cytosol, and mitochondria and helps plants detoxify H_2_O_2_ by converting it into H_2_O [87,88]. Chaperonin 60 kDa protein (Spot 29), was upregulated in *PP2C9TL* under low N, these chaperonins which help and guide cells in the correct folding of proteins both in normal conditions [89] and under stress [90]. The upregulation of chaperonin 59.7 kDa protein was also reported in microbes in the low N condition [91].

## 4. Materials and Methods 

### 4.1. Plant Materials and Growth Conditions

These research experiments were carried out at the greenhouse of the experimental farm of Fujian Agriculture and Forestry University, Fuzhou, Fujian province, China during the rice growing season (April to October 2017). The region has a humid subtropical climate with mean temperatures ranging from 15 to 34 °C in 2017. Two isogenic rice lines of the wild-type, Kitaake (*Oryza sativa* L. ssp. *Japonica*) and its counterpart, the *PP2C9* transgenic line (*PP2C9TL*), in which the *PP2C9* 9 gene (LOC8058897) has been transformed from sorghum bicolor, overexpressing the protein phosphatase 2C 9 protein (XP_002456624.1) were used as research materials. Rice seeds were soaked in distilled water at 25 °C for 24 h and later incubated at 37 °C for 48 h. The germinated seeds of similar size were sown to obtain uniform seedlings which were transplanted to plastic buckets sized in 0.3 m top and 0.23 m bottom diameter with four hills per pot. The sandy loam soil was mixed well to make it uniform before being used for potting with available N (190.6 mg kg^−1^), phosphorus (126.6 mg kg^−1^), and potassium (201.6 mg kg^−1^). The recommended level of N in Fuzhou (225 kg ha^−1^) and half of this amount (112.5 kg ha^−1^) were set as the treatment and control, respectively. Phosphorus was used as the base fertilizer and potassium as the top dressing, at the rate of 112.5 kg ha^−1^ (P_2_O_5_), and 180 kg ha^−1^ (K_2_O) converted to the amounts per barrel, respectively.

### 4.2. Transgenic Line Generation

The full length ORF of PP2C9 (GenBank accession no. LOC8058897) was cloned into the p3301 vector under the control of cauliflower mosaic virus promoter (CaMV 35S) including a FLAG Octapeptide tag. The agrobacterium-mediated transformation of rice immature embryos from mature seeds was used following the protocol of a past paper [92]. Transformed cells obtained from these tissues were selected on the basis of hygromycin resistance, and transgenic plants were finally regenerated. The transgenic plants were screened and confirmed with a Flag-tag assay.

### 4.3. Physiological Measurement

The leaves were sampled from both genotypes at tillering, 5, 10, 15, 20, 25, and 30 days after flowering (DAF). To collect samples, four plants were collected from plastic buckets and the leaves were water-washed and separated. Collected leaf samples were dried in an oven at 105 °C for 30 min, and afterward, dried at 80 °C for 48 h until constant weight. Dry weight was recorded as described by Yoshida [93]. Chlorophyll content from the leaves was measured according to Xiong et al. [94], by using a SPAD-502 chlorophyll meter (Konica-Minolta, Osaka, Japan). The readings were taken in the morning between 9:00 and 10:30 a.m. from flag leaves in four replications. The leaf samples were digested with H_2_SO_4_-H_2_O_2_ and the leaf N concentration was determined using the Kjeldahl method according to Sáez-Plaza et al. [95]. Sampling was done in three replications and SPSS software was used for the analysis of variance (ANOVA) by the least significant difference at *p* < 0.05 (LSD_0.05_). 

### 4.4. Protein Sample Preparation

Leaf samples were collected at tillering, 10 DAF, and 30 DAF from *WT* and *PP2C9TL* plants under control and low N conditions. Leaf samples were washed, freeze-dried with liquid nitrogen, and saved at −80 °C. Protein extraction from leaves was performed by following the protocol of Li et al. [96]. Concisely, 5 g of frozen leaf samples at −80 °C was ground to powder along with polyvinylpyrrolidone (PVP) and liquid nitrogen by using mortar and pestle. The obtained fine powder was mixed in precooled acetone containing 10% TCA (Trichloroacetic acid) and 0.07% β-mercaptoethanol and kept at −20 °C overnight. The sample was centrifuged at 16,000 *g* for 30 min at 4 °C. The supernatant was discarded and obtained pellet was dissolved in 20mL precooled 100% acetone containing 0.07% β-mercaptoethanol, slightly vortexed and kept at −20 °C for 3 h., a transparent supernatant was obtained by repeating this step many times. The precipitate was freeze-dried under vacuum to powder. The obtained powder was dissolved in a buffer (pH 8.0) containing 8 mol/L thiourea, 4% CHAPS (3-[(3-cholamidopropyl) dimethylammonio]-1-propanesulfonate), 40 mmol/L Tris, and 65mmol/L DTT (dithiothreitol). Bradford method is used to measure the protein concentration by using BSA (Bovine Serum Albumin) as a standard [97].

### 4.5. Two-Dimensional Gel Electrophoresis

Based on the physiological response information of the two rice genotypes exposed to the nitrogen treatment at the pot, 2DE of proteins was performed to separate the leaf proteins at 10 DAF using the isoelectric focusing (IEF) strip gels (17 cm, pH 4–7; Bio-Rad, Hercules, CA, USA) for the first dimension. For IEF, an immobiline dry strip gel was rehydrated at 20 °C for 14 h and 1.3 mg protein was loaded in each strip. Protean IEF apparatus (Bio-Rad, USA) was used to do IEF. The voltages applied were at a gradient of 500 V for 1 h; gradient of 1000 V for 2 h; gradient of 8000 V for 3 h; held at 8000 V for 3 h; and then at a gradient of 1000 V for 24 h, as mentioned in [45]. After completing the IEF, the strips were further exposed to an equilibration buffer. Equilibration buffer I (65 mM DTT) was used for 15 min shaking. Iodoacetamide (2.5% (*w*/*v*) was used and kept shaking for 15 min. The second-dimensional separation was carried out on SDS-PAGE containing 12% (*v*/*v*) polyacrylamide gels in 2D-Electrophoresis SDS-PAGE Apparatus (GE) at 15 mA current per gel until completion of electrophoresis. The gels were stained with Colloidal Coomassie Blue G-250 for 12 h followed by destaining. GE Image scanner III was used to scan the obtained protein gels and Imagemaster 5.0 software was used to identify comparative protein spots.

### 4.6. In-Gel Protein Digestion

Differential protein spots were cut from the gel and washed with deionized water and rehydrated with acetonitrile and ammonium bicarbonate as mentioned in [45]. The gel was destained twice with 100 μL of acetonitrile (50%)/100 mM ammonium bicarbonate (50%) for 10 min and then dehydrated with 100% acetonitrile. Lastly, samples were digested with 20 μL of trypsin (12.5 μg mL^−1^ 50 mM ammonium bicarbonate) for 30 min on the ice and then incubated at 37 °C overnight. The 20% acetonitrile and 1% formic acid were added to the gel and centrifuged to obtain the supernatant which later used for LC-ESI-MS/MS analysis.

### 4.7. LC-ESI-MS/MS Analysis and Protein Identification

The LC-ESI-MS/MS analysis was performed by the protocol of Li, Li, Muhammad, Lin, Azeem, Zhao, Lin, Chen, Fang, and Letuma [96]. High performance liquid chromatography: Thermo Scientific Accera System; Chromatographic Column: Bio Basic C18 Column (100 × 0.18 mm, the particle size: 5 μm). Loading quantity of sample: 10 uL. Mobile phase: Solvent A was 0.1% HCO_2_H mixed in water, and Solvent B was 0.1% HCO_2_H mixed in CH_3_CN. Gradient was held at 2% solvent B for 2 min and increased linearly up to 90% Solvent B for 60 min. The peptides were eluted from a C18 column at a flow rate of 160 μL min^−1^ and then electro-sprayed directly into an LTQ mass spectrometer using a spray voltage of 3.5 kV and a constant capillary temperature of 275 °C. Data retrieved from data-dependent MS/MS scanning mode. Mass spectrometry analysis of the raw data obtained in Proteome Discoverer 1.2 relative quantitative analysis software and database retrieval was performed through UniProt database (http://www.uiprot.org/) using the *Oryza sativa* Fasta protein libraries 2.6 Software Analysis.

### 4.8. Confirmation of Important Protein 14-3-3 by Western Blotting Analysis 

To perform Western blotting, the primary antibody for 14-3-3 (Catalog No. 51-0700) and Flag DYKDDDDK Tag Monoclonal Antibody (FG4R) (Catalog No. MA1-91878) were obtained from (Thermo Fisher Scientific, Waltham, MA, USA). The proteins for western blotting were transferred to tubes containing 300 µL SDS loading buffer and boiled at 95 °C for 10 min. Each sample was separated by SDS-PAGE and blotted on nitrocellulose membranes. The membranes were blocked with 5% bovine serum albumin (BSA) in phosphate buffered saline with tween 20 (PBST), after that, incubated with primary antibodies (rabbit antisera to 14-3-3 or Flag) with dilutions following manufactures instructions. Horseradish peroxidase (HRP) conjugated goat anti-rabbit antibodies were used as the secondary antibodies. Antibody-tagged protein spots were detected by 3, 3′-diaminobenzidine (DAB).

### 4.9. Co-Immunoprecipitation (Co-IP) Assay

The plant cell lysate in 1 ml of western and IP buffer [20 mM Tris (pH 7.5), 150 mM NaCl, 1% Triton X-100] were incubated with the lab-preserved rabbit antisera polyclonal Flag-tag antibody (Thermo Fisher Scientific, USA) dilutions following the manufacturer’s instructions for overnight at 4 °C. The next day samples incubated together with protein G Agarose (PGS) beads for 3 to 5 h of slowly shaking at 4 °C then centrifuged (1500× *g*) to collect proteins, and were then washed with PBS buffer. After 5 times centrifugation and washing, the protein sample was transferred to a new Eppendorf tube and SDS-PAGE was performed, as mentioned above.

### 4.10. Statistical Analysis

Functional characterization of differential proteins was done by using, Web gene ontology annotation plot (WEGO), and Kyoto encyclopedia of genes and genomes (KEGG) by Ye et al. [98]. Origin 8.0 was used for graphical analysis [99] and SPSS was used for statistical analysis [100].

## 5. Conclusions

Nitrogen (N)-based fertilizer is a key factor for crop productivity. To maximize yields, farmers extensively use the nitrogenous fertilizers, however, a limited amount of N is utilized by crop plants and the remaining amount causes severe environmental pollution. The development of genotypes with high nitrogen utilization efficiency (NUE) which can grow efficiently and sustain yields in low N conditions are imperative for sustainable agriculture. In the present study, two isogenic lines wild-type, kitaake, and *PP2C9TL* overexpressing the protein phosphatase gene were used for comparative proteomics to identify candidate genes related with high NUE. This study confirmed the differential expression of the protein in the two isogenic lines. In the leaf proteome, 30 protein spots were differentially expressed between two genotypes under low N level, and mostly, the proteins were involved in energy and metabolism. This provides evidence about the connection between N and carbon metabolism. However, the 14-3-3 protein was reported to reduce N- and C-containing compounds and negatively affect the TCA cycle in low N conditions. In this study, the *PP2C9TL* overexpressed *PP2C9* gene downregulated 14-3-3 to enhance NR activity. The proteomics analysis confirmed that *PP2C9TL* exhibits a higher energy level (TCA cycle) than *WT* due to the downregulation of 14-3-3 protein. Physiological and proteomics studies showed that overexpression of protein phosphatase in *PP2C9TL* significantly increased the NUE. Physiological study coupled with proteomic analysis provides a potential basis for genetic-based selection of N-related genotypes to increase N efficiency in cereal crops.

## Figures and Tables

**Table 1:** Differential expression of leaf proteins between *WT* and *PP2C9TL* at 10 DAF under low N conditions.

SN ^a^	AN ^b^	Protein Name	Functional Characterization	Subcellular Location	Score ^c^	MW (kDa)/pI ^d^	MP ^e^	CT ^f^
**Energy Metabolism**								
3	A6N1P5.1	Ribulose-1,5-bisphosphate carboxylase/oxygenase	Energy (photosynthesis)	Chloroplast	69	30.36/5.22	12	UR
10	P93431-1	RuBisCO	Energy (photosynthesis)	Chloroplast	45	51.42/5.16	4	UR
20	Q6ZG90	ATP synthase	Energy	Mitochondria	46	27.3/6.93	4	UR
7	Q8S6Z1.1	ATP synthase subunit alpha	ATP energy	Mitochondria	136	29.30/5.33	9	UR
2	A3C6G9.1	Glycine cleavage system H protein	Energy	Mitochondria	108	17.3/5.03	11	UR
24	A1YQK1	Malate dehydrogenase	TCA cycle	Cytoplasm	155	36.5/6.33	6	DR
**Photosynthesis**								
26	Q9FYX8	Phosphoenolpyruvate carboxylase	Photosynthesis	Cytosol	98	105.2/5.73	8	DR
30	E9KIQ8	Photosystem II CP47	Photosynthesis	Chloroplast	89	53.24/5.67	7	UR
22	Q652K1	Stay-green protein	Photosynthesis	Chloroplast	234	121.32/4.66	13	DR
**Carbohydrate Metabolism**								
1	Q6Z8F4.1	Phosphoribulokinase	Calvin cycle	Chloroplast	43	44.83/6.02	2	DR
5	Q53P94.1	Fructose-bisphosphate aldolase	Glycolysis	Chloroplast	138	14.24/7.50	4	DR
9	Q6EQ16.1	ATP-dependent 6-phosphofructokinase	Glycolysis	Chloroplast	121	52.7/7.65	10	DR
14	Q0INM3.1	Beta-galactosidase 15	Carbohydrate metabolism	Cytosol	269	100.8/5.95	16	DR
6	Q84JG8	Chloroplast sedoheptulose-1,7-bisphosphatase	Calvin cycle	Chloroplast	75	42.21/5.84	6	UR
**Nitrogen Metabolism**								
23	Q6ZHH7	Nitrate reductase	Nitrogen metabolism	Chloroplast	271	97.79/534	13	UR
19	P14655	Glutamine synthetase	Nitrogen metabolism	Chloroplast	67	47.08/5.96	7	UR
**Cell Growth and Division**								
18	Q10PE7	Deoxymugineic acid synthase 1	Growth	Cytosol	129	33.6/7.5.41	13	UR
13	Q7XE16	Cell division cycle 48	Cell division	Cell membrane	134	88.8/5.23	15	DR
**Signaling**								
15	Q06967	14-3-3 protein GF 14-B	Defense	Nucleus	114	18.27/4.46	21	DR
27	Q18PR8	Beta subunit 2 of SnRK1	signaling	Membrane	46	32.76/5.44	9	DR
25	Q6K1U4	Probable protein phosphatase 2C 16	Phosphatase	Nucleus	94	56.21/4.17	19	UR
**Transcription**								
11	Q6IER3	WRKY 8	Transcription	Nucleus	357	30.36/7.07	20	DR
8	E5RQA1	GHD 7	Transcription	Nucleus	112	26.83/7.23	14	DR
21	Q84MM9	Monoculm protein 1	Transcription	Nucleus	123	48.5/7.54	18	UR
**Storage and Structural Protein**								
29	Q8H903	Chaperonin 60 kDa protein	Protein synthesis, folding	Cytosol	336	60.8/5.78	12	DR
**Defense**								
17	Q943K7	HSP 70	Defense	Nucleus	90	71.23/5.21	7	UR
28	Q5QT28	Remorin 1 protein/Hsp20	Defense	Nucleus	44	22.4/537	6	UR
16	Q9FE01	L-ascorbate peroxidase	Defense	Chloroplast	75	28.49/5.36	14	DR
4	Q6YUZ0	Thaumatin-like protein	Defense	Cytoplasm	97	18.81/4.88	10	DR

Note: ^a^ Protein spot numbers correspond to 2-DE gels shown in Figure 4. ^b^ Accession number according to the UniProt database. ^c^ Score, ions score of identified proteins using rice genome sequence databases. ^d^ Theoretical MW (kDa) and pI values. MW, molecular weight; pI, isoelectric point. ^e^ M.P., number of query matched peptides. ^f^ Changes in the protein spots between PP2C9TL and WT under low N conditions; UR: protein spots upregulated at grain-filling stage (10 DAF), DR: protein spots downregulated at grain-filling stage (10 DAF).

**Table 2:** LC-MS/MS analysis of the Co-immunoprecipitation of *PP2C9TL*.

AN ^a^	Protein Name ^b^	Functional Characterization	Subcellular Location	Score ^c^	MW (kDa)/pI ^d^
**Energy Metabolism**					
LOC_Os12g10580.1	Ribulose bisphosphate carboxylase large chain	Energy (photosynthesis)	Chloroplast	371.7	56/8.9
LOC_Os10g21268.1	Ribulose bisphosphate carboxylase large chain	Energy (photosynthesis)	Chloroplast	683.23	53.7/7.03
LOC_Os06g39740.1	ATP synthase subunit beta	Energy	Mitochondria	576.28	54.2/5.5
LOC_Os06g45120.1	ATP synthase	ATP energy	Mitochondria	178.54	68.4/5.34
LOC_Os10g17280.1	ATP synthase gamma chain	Energy	Mitochondria	16.83	35.2/7.03
LOC_Os04g16740.1	ATP synthase subunit alpha	ATP energy	Mitochondria	279.38	55.6/6.25
LOC_Os03g17070.1	ATP synthase B chain chloroplast precursor	ATP energy	Mitochondria	39.69	22.7/5.85
LOC_Os10g37180.1	Glycine cleavage system H protein	Energy	Mitochondria	50.25	17.4/5.03
LOC_Os03g56280.1	Malate dehydrogenase	TCA cycle	Cytoplasm	146.16	37/7.94
**Photosynthesis**					
LOC_Os08g27840.1	Phosphoenolpyruvate carboxylase	Photosynthesis	Cytosol	260.03	110/5.8
LOC_Os04g16874.1	Photosystem II 44 kDa reaction center protein	Photosynthesis	Chloroplast	94.42	44.7/6.6
LOC_Os03g21560.1	Photosystem II 11 kD protein	Photosynthesis	Chloroplast	21.05	17.6/9.91
LOC_Os07g05360.1	Photosystem II 10 kDa polypeptide, chloroplast	Photosynthesis	Chloroplast	22.11	14.2/9.81
LOC_Os02g24634.1	Photosystem II D2 protein	Photosynthesis	Chloroplast	64.09	39.6/5.4
LOC_Os06g39708.1	Photosystem II P680 chlorophyll a Apoprotein	Photosynthesis	Chloroplast	161.07	56.2/6.64
**Carbohydrate Metabolism**					
LOC_Os02g47020.1	Phosphoribulokinase	Calvin cycle	Chloroplast	266.6	44.8/6.02
LOC_Os04g16680.1	Fructose-1,6-bisphosphatase	Glycolysis	Chloroplast	256.8	42.2/6.09
LOC_Os11g07020.1	Fructose-bisphosphate aldolase isozyme	Glycolysis	Chloroplast	403.24	42/6.8
LOC_Os01g64660.2	Fructose-1,6-bisphosphatase	Carbohydrate metabolism	Cytosol	127.72	37/5.77
LOC_Os08g03290.1	Glyceraldehyde-3-phosphate dehydrogenase	Carbohydrate metabolism	Cytosol	97.76	36.4/7.11
**Nitrogen Metabolism**					
LOC_Os02g50240.1	Glutamine synthetase	Nitrogen metabolism	Chloroplast	134.89	39.2/5.73
LOC_Os05g04220.1	Nitrogen regulatory protein P-II	Nitrogen metabolism	Chloroplast	5.02	22.7/9.91
LOC_Os02g52730.1	Ferredoxin-nitrite reductase	Nitrogen metabolism	Chloroplast	3.35	72.4/8.29
LOC_Os04g01590.1	Arginase	Nitrogen metabolism	Mitochondria	14.54	36.9/5.3
LOC_Os09g28050.1	Asparate aminotransferase	Nitrogen metabolism	Chloroplast	19.62	50.6/6
**Cell Growth and Division**					
LOC_Os10g30580.1	Cell division	Growth	Cytosol	42.5	89.8/5.21
LOC_Os02g58790.1	Cell division inhibitor	Growth	Cytosol	6.35	38.8/9.16
**Signaling**					
LOC_Os03g50290.1	14-3-3 protein	Defense	Nucleus	112.53	29.2/4.88
LOC_Os04g38870.3	14-3-3 protein	Signaling	Membrane	88.34	29.8/4.81
LOC_Os08g33370.2	14-3-3 protein	Phosphatase	Nucleus	74.19	28.8/4.84
LOC_Os05g11550.1	Serine/threonine protein phosphatase 5	Growth	Cytosol	5.86	54.4/6.02
LOC_Os07g32380.1	Protein phosphatase 2C	Phosphatase	Nucleus	4.9	20/8.18
LOC_Os09g06230.1	Serine/threonine-protein kinase 16	Phosphatase	Nucleus	3.21	25.5/4.7
LOC_Os04g56450.1	Protein phosphatase 2C	Signaling	Membrane	1.89	30.6/5.15
LOC_Os09g33790.1	SnRK1-interacting protein 1	Signaling	Membrane	2.95	109/7.15
LOC_Os02g38300.1	SNF7 domain-containing protein	Signaling	Membrane	2.69	25.5/4.7
**Transcription**					
LOC_Os03g55164.1	WRKY4	Transcription	Nucleus	2.47	14.8/7.28
**Storage and structural protein**					
LOC_Os06g09679.2	Chaperonin	Protein synthesis, folding	Cytosol	81.66	26.3/8.02
LOC_Os10g41710.1	Chaperonin, putative expressed	Protein synthesis, folding	Cytosol	5.18	21.1/8.92
LOC_Os09g26730.1	Chaperonin	Protein synthesis, folding	Cytosol	70.68	69.1/6.37
**Defense**					
LOC_Os10g07210.1	Hsp20/alpha crystallin family protein	Defense	Nucleus	2.26	18.6/8.2
LOC_Os06g37150.1	L-ascorbate oxidase precursor	Defense	Chloroplast	2.9	27.4/7.65
LOC_Os07g49400.2	Cytosolic Ascorbate Peroxidase	Defense	Chloroplast	68.37	25.2/5.71
LOC_Os12g43440.1	Thaumatin putative,	Defense	Cytoplasm	9.64	22.4/5.5
LOC_Os07g47510.1	Stress-related protein,	Defense	Nucleus	2.38	27.4/7.65
LOC_Os09g29200.1	Glutathione S-transferase,	Defense	Cytoplasm	134.49	25.2/5.71
LOC_Os04g45070.1	Remorin	Defense	Nucleus	45.89	22.4/5.5

## Supplementary Materials

Supplementary materials can be found at http://www.mdpi.com/1422-0067/19/9/2827/s1. Figure S1 Phenotypic response of *WT* and *PP2C9TL* under different levels of N at heading stage. Figure S2: Plant height of *WT* and *PP2C9TL* under different levels of N at Tillering, Heading, and Maturity stage. Figure S3: Representative 2-DE gel electrophoresis images from the leaf total proteins of WT and PP2C9TL, sampled at 10 DAF under control N treatment. Figure S4: Representative 2-DE gel electrophoresis images from the leaf total proteins of WT and PP2C9TL, sampled at 10 DAF under low N treatment. Figure S5: Representative 2-DE gel electrophoresis images of differentially expressed proteins of WT and PP2C9TL leaves, sampled at 10 DAF under low N treatment. Figure S6: Representative 2-DE gel electrophoresis images of differentially expressed proteins of WT and PP2C9TL leaves, sampled at 10 DAF under control N treatment.
