# Supplementary material for: Protein Phosphatase (PP2C9) Induces Protein Expression Differentially to Mediate Nitrogen Utilization Efficiency in Rice under Nitrogen-Deficient Condition
Source: Int J Mol Sci. 2018 Sep 19;19(9):2827. doi: 10.3390/ijms19092827 (PMC6163212; doi:10.3390/ijms19092827)
Supplement: Supplementary file 1 [file ijms-19-02827-s001.pdf]

Protein Phosphatase (*PP2C9*) Induces Protein Expression Differentially to Mediate Nitrogen Utilization Efficiency in Rice under Nitrogen-Deficient Condition

Muhammad Waqas [1,2,*], Shizhong Feng [3], Hira Amjad [2,3], Puleng Letuma [2,3], Wenshan Zhan [3], Zhong Li [1,2], Changxun Fang [1,2,3], Yasir Arafat [2,3], Muhammad Umar Khan [2,3], Muhammad Tayyab [1,2], Wenxiong Lin [1,2,3,*]

[1] Key Laboratory for Genetics, Breeding and Multiple Utilization of Crops, Ministry of Education/College of Crop Sciences, Fujian Agriculture and Forestry University, Fuzhou 350002, China; lizhong021@126.com (Z.L.); fcx007@fafu.edu.cn (C.F.); tyb.pk@hotmail.com (M.T.)

[2] Fujian Provincial Key Laboratory of Agroecological Processing and Safety Monitoring, College of Life Sciences, Fujian Agriculture and Forestry University, Fuzhou 350002, China; heraamahmood@yahoo.com (H.A.); pulengletuma@yahoo.com (P.L.); arafat_pep@yahoo.com (Y.A.); umar.khan018@yahoo.com (M.U.K.)

[3] Key Laboratory of Crop Ecology and Molecular Physiology (Fujian Agriculture and Forestry University), Fujian Province University, Fuzhou 350002, China; M18120821563@163.com (S.F.); 1170525027@fafu.edu.cn (W.Z.)

* Correspondence: waqasjutt_19@yahoo.com (M.W.); lwx@fafu.edu.cn (W.L.)

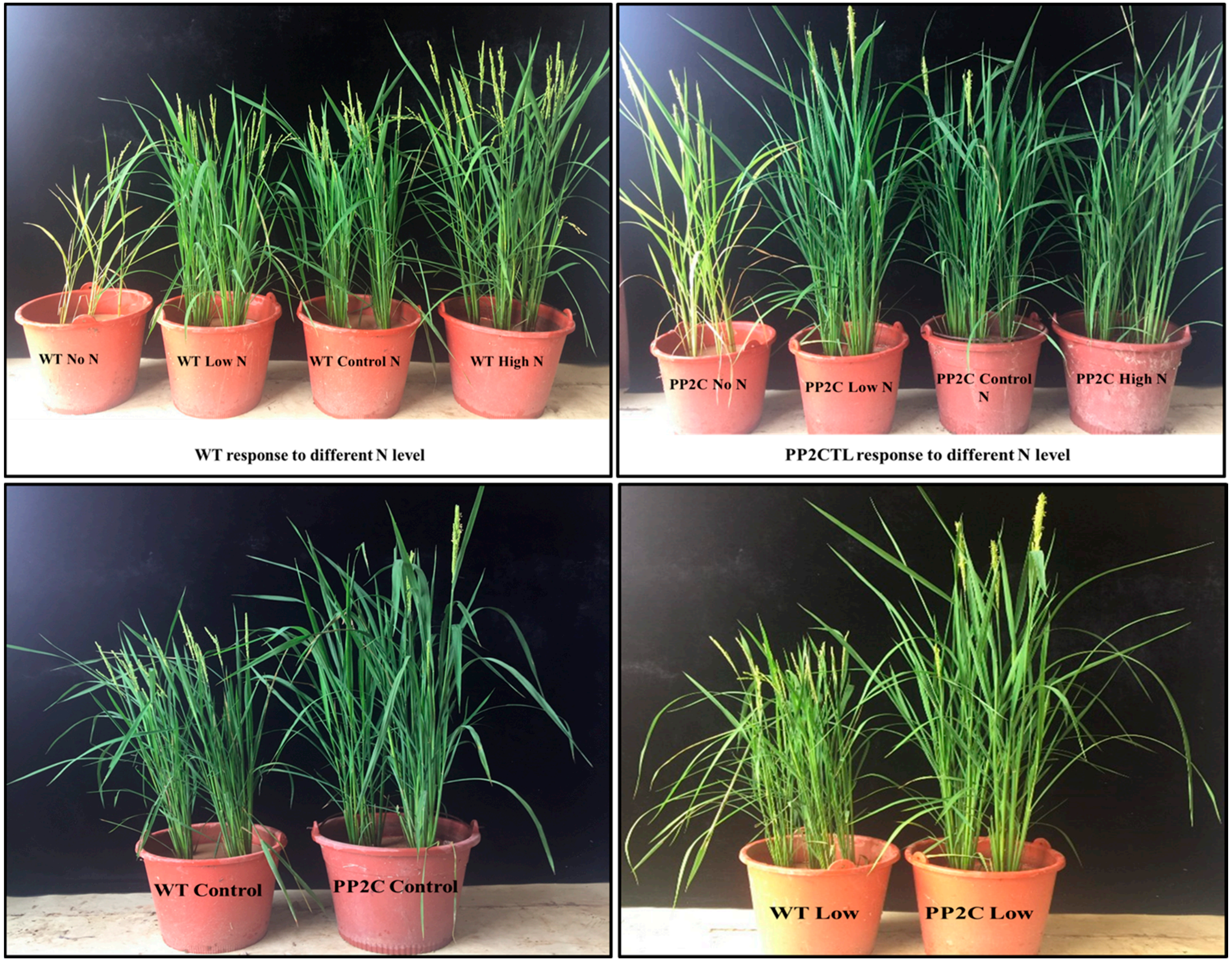

Figure S1: Phenotypic response of *WT* and *PP2C9TL* under different levels of N at heading stage.

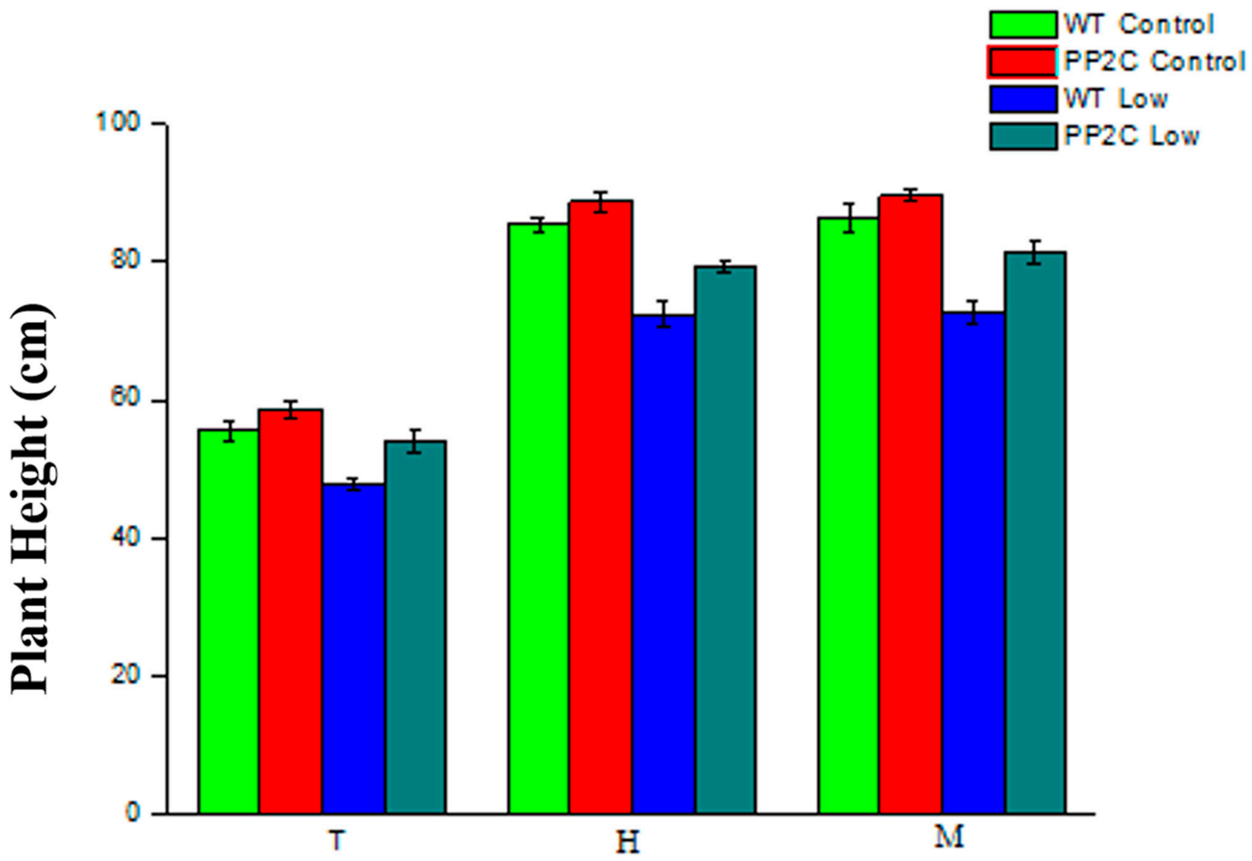

Figure S2: Plant height of *WT* and *PP2C9TL* under different levels of N at Tillering, Heading and Maturity stage.

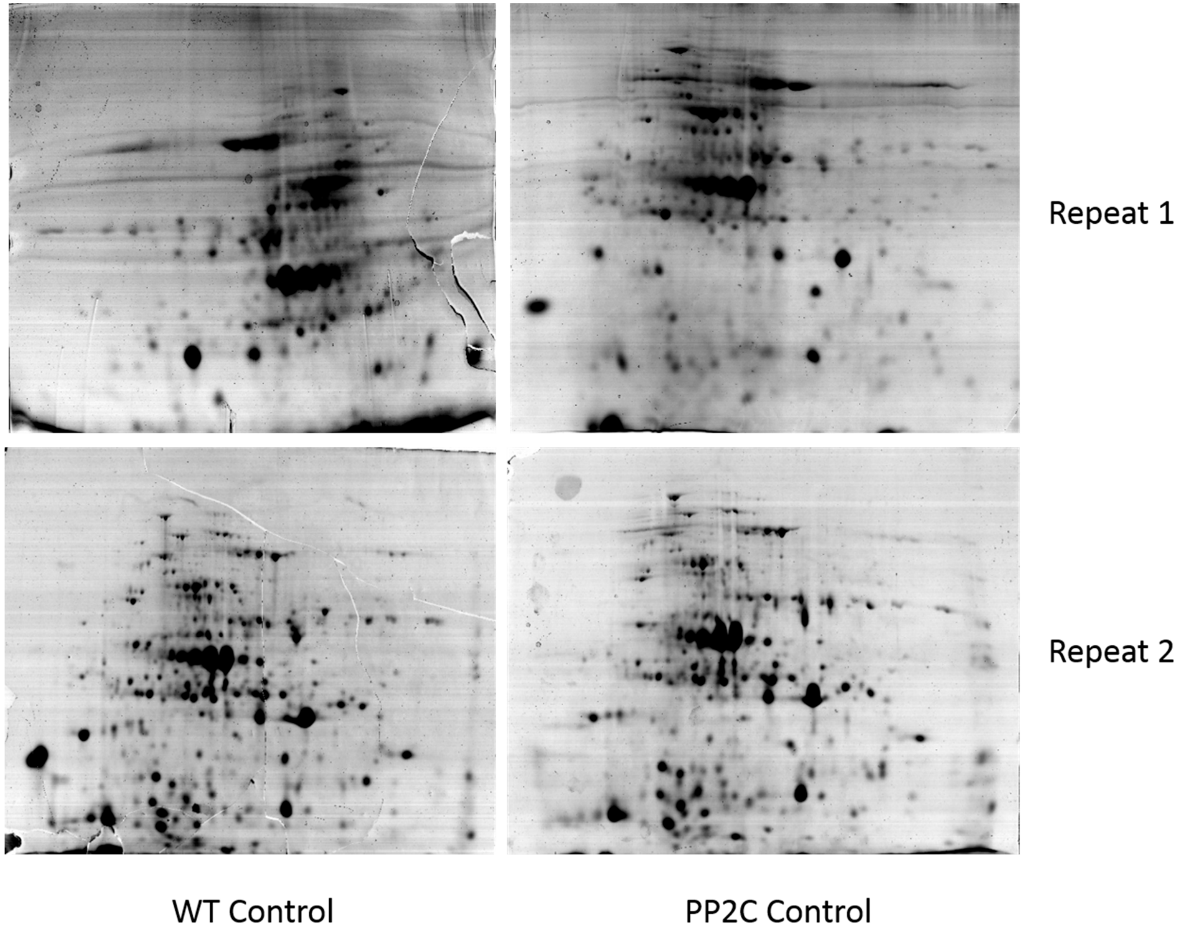

Figure S3: Representative 2-DE gel electrophoresis images from the leaf total proteins of WT and PP2C9TL, sampled at 10 DAF under control N treatment.

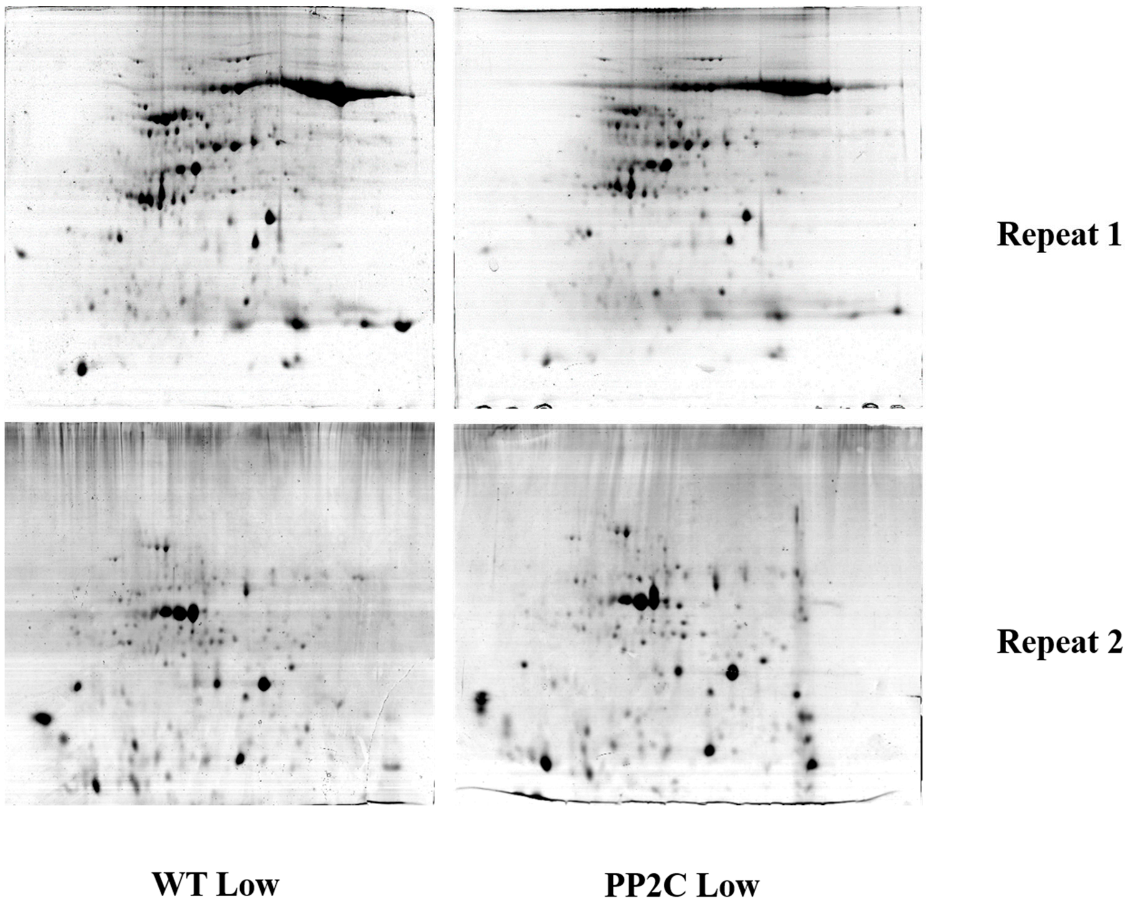

Figure S4: Representative 2-DE gel electrophoresis images from the leaf total proteins of WT and PP2C9TL, sampled at 10 DAF under low N treatment.

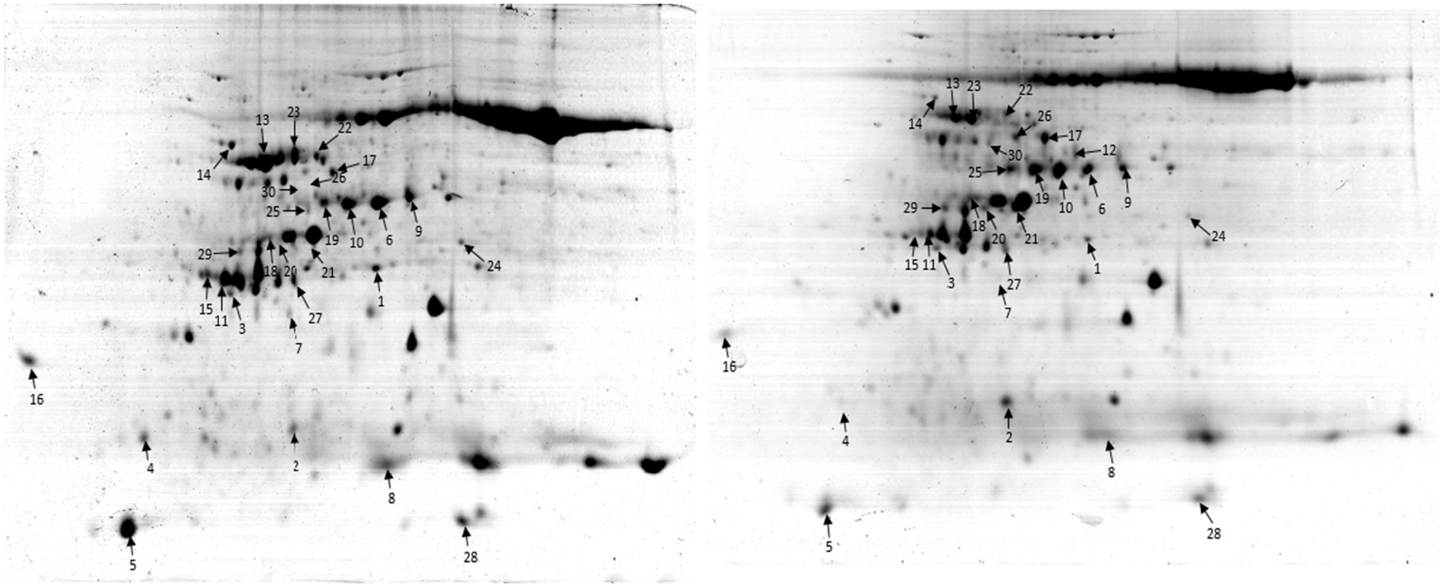

WT Low **PP2C Low**

Figure S5: Representative 2-DE gel electrophoresis images of differentially expressed proteins of WT and PP2C9TL leaves, sampled at 10 DAF under low N treatment.

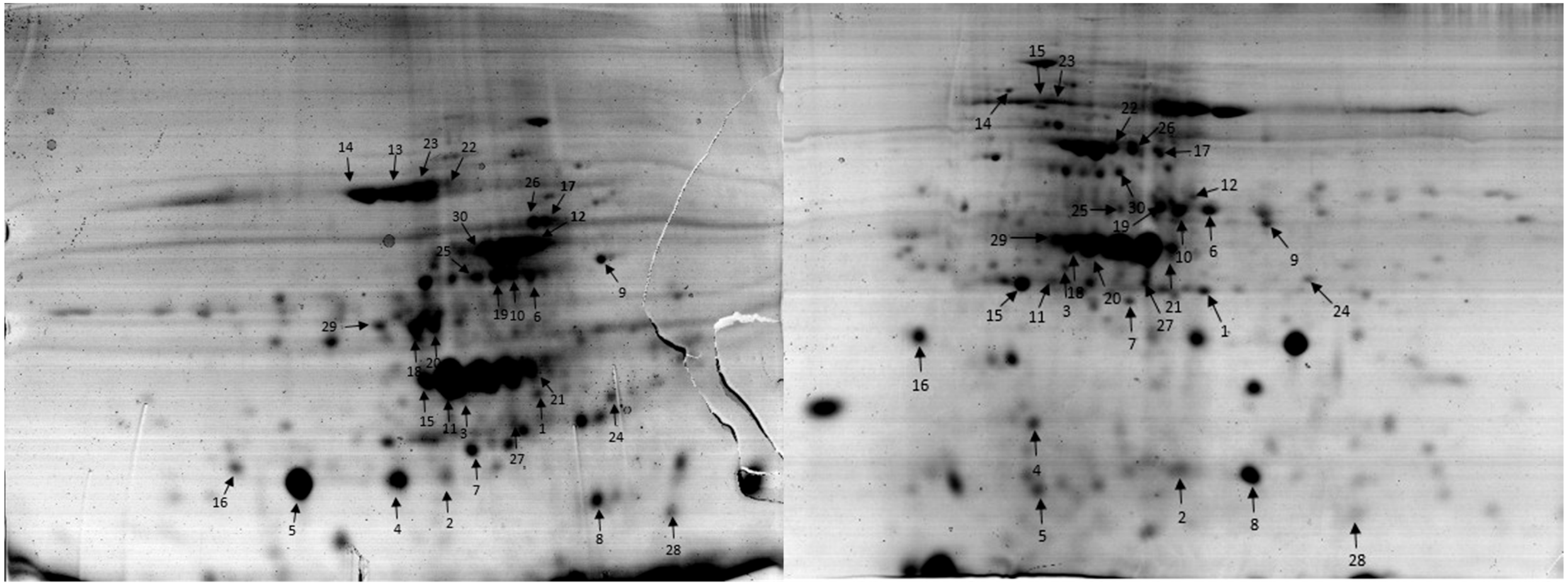

WT Control **PP2C Control**

Figure S6: Representative 2-DE gel electrophoresis images of differentially expressed proteins of WT and PP2C9TL leaves, sampled at 10 DAF under control N treatment.
